# Adherence to iron supplement intake during pregnancy and associated factors in Ethiopia: Further analysis of a national population‐based study

**DOI:** 10.1002/fsn3.3503

**Published:** 2023-06-15

**Authors:** Takele Gezahegn Demie, Getachew Tilahun Gessese, Berhanu Teshome Woldeamanuel, Tolesa Diriba Biratu, Simegnew Handebo

**Affiliations:** ^1^ School of Public Health Saint Paul's Hospital Millennium Medical College Addis Ababa Ethiopia

**Keywords:** adherence, associated factors, Ethiopia, iron supplement intake, pregnancy, pregnant women

## Abstract

Iron deficiency during pregnancy is a risk factor for anemia, preterm delivery, and low birth weight. Poor adherence to iron supplement intake remains a problem in many countries including Ethiopia. This analysis aimed at determining the proportion of adherence to iron supplement intake and its associated factors among pregnant women in Ethiopia. We used the data from the 2019 Ethiopia Mini Demographic and Health Survey (EMDHS), which is a cross‐sectional and nationally representative survey. A weighted sample of 3927 pregnant women was included in the study. Bivariate and multivariable binary logistic regression analyses were performed to identify factors associated with adherence to iron supplement intake. Adjusted odds ratio (AOR) with a 95% confidence interval (CI) and *p*‐value <.05 were used to declare statistical significance. Our analysis revealed that out of 2356 (60.0%) pregnant women who took iron supplements during their most recent pregnancy, only 417 (17.7%; 95% CI: 0.162–0.193) adhered to the WHO‐recommended iron intake for 90 days or more. The subnational regions, level of education, literacy, the timing of first antenatal care booking, and past place of delivery were significantly associated with iron supplement intake. Interventions to enhance the uptake of iron supplementation better focus on improving women's education and literacy, early initiation and frequency of ANC visits, and institutional delivery. Raising community awareness through educating pregnant women is also recommended to improve adherence to iron supplement intake.

## Graphical abstract

The results indicate very low adherence to iron compared to the World Health Organization's iron supplementation recommendations among pregnant women. While respondents’ subnational region, highest educational level, and last place of delivery were positively associated with the outcome variable, literacy and timing of first ANC booking were negatively associated with the outcome variable. These results underscore the need for increased efforts to improve the uptake of iron supplementation for pregnant women in Ethiopia focusing on interventions like early initiation and frequent antenatal care visits, institutional delivery, and raising community awareness through educational programs for pregnant women are recommended to improve adherence to iron intake.

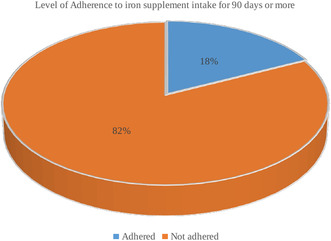

## 1 INTRODUCTION

Nutritional anemia is the leading public health problem among pregnant women worldwide (Digssie Gebremariam et al., 2019; Tarekegn et al., 2019), particularly in developing countries. It is estimated that about 42% of pregnant women are anemic worldwide and at least half of this anemia burden is assumed to be due to iron deficiency (Solomon et al., 2021; WHO & 1000 DAYS, 2012). Anemia directly causes one in five maternal deaths worldwide (Khanal et al., 2014). To prevent maternal anemia, puerperal sepsis, low birth weight, and premature birth, the World Health Organization (WHO) recommends iron supplementation from the second trimester of pregnancy until 45 days after delivery (Haider et al., 2013; World Health Organization, 2012). One of the Sustainable Development Goals (SDGs) is to reduce anemia, which is acknowledged as being crucial to women's health (WHO & 1000 DAYS, 2012; World Health Organization, 2020). To achieve the 2025 global nutrition target, anemia in women of reproductive age must be reduced by 50% (WHO & 1000 DAYS, 2012). (WHO & 1000 DAYS, 2012).

Increased blood volume, a lack of iron and folic acid, or poor adherence all increase the risk of anemia in pregnant women. In Sub‐Saharan Africa (SSA), iron deficiency anemia during pregnancy is a significant public health problem and is associated with severe adverse health outcomes (Agegnehu et al., 2019; Ethiopian Public Health Institute (EPHI) [Ethiopia] & ICF, 2021; Gebreamlak et al., 2017; Lyoba et al., 2020). Pregnant women are encouraged to take iron folate (or iron–folic acid (IFA)) supplements throughout pregnancy with optimal adherence (Ethiopian Public Health Institute (EPHI) [Ethiopia] & ICF, 2021; Gebreamlak et al., 2017; Triharini et al., 2018), one of the most cost‐effective strategies to lessen/prevent iron deficiency anemia during pregnancy, low birth weight, and prematurity (Demis et al., 2019; Digssie Gebremariam et al., 2019; Gebreamlak et al., 2017; Gebremichael & Welesamuel, 2020; Tarekegn et al., 2019). A key process indicator of the Global Nutrition Monitoring Framework (GNMF) is the prevalence of IFA supplementation among pregnant women (World Health Organization, 2018).

Iron supplementation for at least 90 days during the most recent birth's pregnancy is required for adherence to iron supplement intake (Ba et al., 2019) although Agegnehu et al used different definitions (Agegnehu et al., 2019; Digssie Gebremariam et al., 2019). The WHO recommends daily oral IFA supplementation as part of prenatal care (ANC) to avoid anemia, although utilization is still poor in SSA, especially in Ethiopia (Mekonnen et al., 2021). Given the high prevalence of iron deficiency anemia in SSA, poor adherence to iron supplements has reduced their efficacy in lowering maternal anemia (Kiwanuka et al., 2017). An extensive study of pregnant women in SSA found that 28.7% of them adhered to the recommendation to take iron supplements for at least 90 days, with adherence rates ranging from 1.4% in Burundi to 73.0% in Senegal (Ba et al., 2019).

In Ethiopia, the proportion of women who have taken iron supplements for at least 90 days climbed from 5% in 2016 to 11% in 2019, which is by far below the recommendation. The percentage of women who did not take any iron supplements reduced from 58% to 40% over the same period (Ethiopian Public Health Institute (EPHI) [Ethiopia] & ICF, 2021). Furthermore, local research in Dire Dawa, Eastern Ethiopia's public hospitals, reported a variety of findings ranging from 10.5% (Gebremichael et al., 2019) to 67.6% (Mekonnen et al., 2021) and 71.8% (Solomon et al., 2021).

Numerous studies have shown that factors such as a woman's age (Ba et al., 2019; Gebremichael & Welesamuel, 2020; Titilayo et al., 2016), education level (Agegnehu et al., 2019; Boti et al., 2018; Gebreamlak et al., 2017; Tarekegn et al., 2019; Tegodan et al., 2021; Titilayo et al., 2016), number of pregnancies (Gebremichael et al., 2019), family size (Agegnehu et al., 2019), number of children (Lyoba et al., 2020), early registration, the frequency and number of antenatal visits (Agegnehu et al., 2019; Ba et al., 2019; Boti et al., 2018; Demis et al., 2019; Digssie Gebremariam et al., 2019; Gebremichael et al., 2019; Getachew et al., 2018; Kiwanuka et al., 2017; Lyoba et al., 2020; Solomon et al., 2021; Tarekegn et al., 2019; Titilayo et al., 2016) are either positively or negatively associated with iron supplement intake during pregnancy. Additionally, the husband's level of education (Agegnehu et al., 2019; Tarekegn et al., 2019), income (Agegnehu et al., 2019), higher wealth status (Ba et al., 2019; Titilayo et al., 2016), number of meals consumed (Lyoba et al., 2020), knowledge regarding anemia (Assefa et al., 2019; Demis et al., 2019; Getachew et al., 2018; Lyoba et al., 2020; Solomon et al., 2021; Tegodan et al., 2021), and distance to health facility (Lyoba et al., 2020) are determinants of iron supplement intake.

Studies that have previously been conducted were based in health facilities and covered particular settings with relatively small samples taken from ANC follow‐up attendants (Agegnehu et al., 2019; Assefa et al., 2019; Boti et al., 2018; Demis et al., 2019; Digssie Gebremariam et al., 2019; Gebremichael et al., 2019; Gebremichael & Welesamuel, 2020; Getachew et al., 2018; Lyoba et al., 2020; Mekonnen et al., 2021; Obsa et al., 2021; Solomon et al., 2021; Tarekegn et al., 2019; Tegodan et al., 2021).

Suboptimal iron supplement adherence remains problematic in many nations, including Ethiopia. Although it is advised that all pregnant women take an iron supplement, there is limited information and scant research on the subject in Ethiopia. What is more, iron supplementation is one of the antenatal care services provided to expectant mothers; therefore, failing to follow WHO recommendations could jeopardize the standard of care.

Therefore, using recently available and representative data from the national census, the current study assessed the proportion of pregnant women in Ethiopia who adhered to iron supplement intake for 90 or more days during their most recent pregnancy and identified sociodemographic and obstetric factors associated with it. The results of this study will also contribute to boosting pregnant women's iron supplement adherence rates.

## 2 MATERIALS AND METHODS

### 2.1 Study setting and data source

A dataset from the 2019 EDHS was used for this study. The 2019 EMDHS was the second EMDHS that Ethiopia's nine regional states, two chartered city administrations, and the country as a whole (apart from the recently established Sidama and Southern Ethiopia Peoples' regions) have implemented. In collaboration with the Federal Ministry of Health (FMoH) and the Central Statistics Agency (CSA), the Ethiopian Public Health Institute (EPHI) was surveyed with technical support from ICF and financial as well as technical support from development partners. The Growth and Transformation Plan (GTP), which is closely associated with the Sustainable Development Goals, set targets for the health sector, and the 2019 EMDHS collects data to measure the progress of those goals (SDGs). The survey, a community‐based cross‐sectional study based on a nationally representative sample, was carried out from March 21 to June 28, 2019, and it offered estimates for both urban and rural areas, as well as at the national and regional levels (Ethiopian Public Health Institute (EPHI) [Ethiopia] & ICF, 2021).

The Institution Review Board of the Demographic and Health Surveys (DHS) Program, ICF International, granted ethical approval. Each participant in the study provided informed consent, and confidentiality was upheld throughout the collection of EMDHS data. Names of persons or household addresses were not included in the data files, nor were any identifiers for respondents, households, or sample communities permitted in any way.

### 2.2 Study population and sampling procedures

Using stratified and two‐stage cluster sampling techniques, the representative EDHS samples were obtained. In the first stage, each region was stratified into urban and rural areas, and 305 enumeration areas (EAs)—93 in urban and 212 in rural—were chosen independently in each sampling stratum with a probability proportional to the size of the EAs. In the second stage of selection, a fixed number of 30 houses per cluster were chosen from the newly created household list with an equal probability of systematic selection. A total of 8885 women participated in the study, of whom 5846 had ever given birth and 3979 had done so in the 5 years before the survey. All women of reproductive age (15–49) who lived in the chosen residences permanently or who had spent the previous night there as visitors were eligible and selected for interviews. After data cleaning, 3927 women of reproductive age who gave birth during the 5 years before the survey and whose iron supplement intake status was measured in the survey were included in the study. This research used individual women datasets. Women who did not have their iron intake status examined were not included in this analysis. The entire EMDHS 2019 report (Ethiopian Public Health Institute (EPHI) [Ethiopia] & ICF, 2021) included a detailed description of the methodological process. The public domain dataset is accessible and may be downloaded from https://dhsprogram.com/data/available‐datasets.

### 2.3 Data extraction

Secondary data from repositories of demographic and health survey studies were used in this study. The Measure DHS program (http://www.measuredhsprogram.com) permitted access to the Ethiopian DHS 2019 data (downloaded in STATA format). We explored, cleaned, and recorded the data after having a better knowledge of the details. A variety of prospective variables, including sociodemographic characteristics, economic factors, and breastfeeding patterns were taken into account in the final analysis.

## 3 STUDY VARIABLES

### 3.1 Outcome variable

In this analysis, the proportion of pregnant women who adhered to taking iron supplements was defined as taking iron for 90 days during the pregnancy of the most recent birth (Ba et al., 2019). Participants in the study would be recorded as “Adherent” if they used iron supplements for at least 90 days during their most recent pregnancy, and “Not Adherent” otherwise.

### 3.2 Independent variables

Based on their theoretical and practical significance as well as their availability in the dataset, sociodemographic factors, obstetrics, and reproductive characteristics were examined in our study. Maternal age in years, place of residence, subnational area, cluster altitude in meters, sex of the household (HH) head, age of the HH head, religion, marital status, maternal education level, literacy status, wealth index, and family size were among the sociodemographic factors. Obstetrics and reproductive characteristics of the respondents included age of women at first birth, births in the last 3 years, number of under‐five children in the HH, total children ever born, number of living children, preceding birth interval, birth order number, ANC checkup during most recent pregnancy, frequency of ANC visits, the timing of first ANC booking, and past place of delivery.

### 3.3 Data analysis and software

The extracted data were analyzed using the computer software, STATA version 14 software. The analysis of the data included both descriptive and inferential statistics. Individual sample weight (v005/1,000,000) was used in all analyses to account for over‐ and under‐sampling; to get reliable statistical estimates by compensating for the unequal probability of selection between strata and the nonresponse rate among study participants. A detailed explanation of the weighting procedure can be found in the EMDHS methodology report (Ethiopian Public Health Institute (EPHI) [Ethiopia] & ICF, 2021). Descriptive analysis was used to analyze the frequency distribution of the data. Cross‐tabulations were performed and included the presentation of data using frequency tables and figures with their description. Bivariate and multivariable binary logistic regression was used to check for the relation of the independent variable with the outcome variable. In bivariate logistic regression, each variable was checked with the outcome variable and those variables with a *p*‐value of less than .20 were selected as candidate variables for the multivariable logistic regression analysis. Multi‐collinearity was checked using the variance inflation factor (VIF) for independent variables used in multivariable logistic regression analysis and the value of all variables in the final model was less than five with a mean VIF of 2.40. The test of goodness of fit was checked using the Hosmer and Lemeshow test (HLT). Both crude and adjusted odds ratios (AOR) with corresponding 95% confidence intervals (CI) were reported. The threshold for statistical significance was set at *p* < .05 and the results were presented in the form of tables, texts, graphs, and maps.

## 4 RESULTS

### 4.1 Sociodemographic characteristics

The sociodemographic details of the study population are shown in Table 1. More than half (54.4%) of the women were in the age category of 20–29. More than two‐thirds (69.6%) of the respondents were rural dwellers and more than one‐third (35.3%) were from the Oromia region. The cluster altitude ranges from 246 to 3246. The majority of respondents (56.4%) came from low altitudes (500–2000 m). Only 307 (13.1%) of the households had a female head, and 1376 (58.4%) of the household heads were in the age category of 25–39. About 45% of women were Orthodox Christian by religion. Nine hundred and eighty‐seven (41.9%) women had no education at all. At the time of the survey, more than half (55.6%) of women could not read at all. In terms of the wealth index, 644 (27.4%) of women were among the wealthiest, but 324 (19.16%) came from the wealth quantal with the poorest wealth levels. Moreover, half of the households (56.6%) had a family of four or fewer. Only 236 (10.0%) women in the age categories of 20–29 have adhered to iron supplement intake during their more recent pregnancies. Similarly, 272 (11.6%), 245 (10.4%) and 155 (6.6%), 369 (15.7%) of women from rural, low, and moderate altitude, and male‐headed households have adhered to iron supplement intake. Only 180 (7.6%) and 129 (5.5%) women from Oromia and Amhara subnational regions have adhered to iron intake. In the same manner, 213 (9.0%) and 145 (6.2%) women who cannot read at all and those who able to read the whole sentence were adhered to iron intake, respectively. Moreover, 138 (5.9%) and 48 (2.0%) women adhered to the richest and poorest wealth index categories, respective sizes. Additionally, 262 (11.2%) women who had less than five family sizes adhered to iron intake during their more recent pregnancies (Table 1).

**TABLE 1:** Sociodemographic and maternal characteristics of the study population, Ethiopia, 2019 (*n* = 2356).

Variables	Description	Adherence to iron tablet intake (weighted)		
		No, *n* (%)	Yes, *n* (%)	Total, *n* (%)
Women age in years	15–19	91 (3.9)	25 (1.1)	116 (4.9)
	20–29	1045 (44.4)	236 (10.0)	1281 (54.4)
	30–39	662 (28.1)	131 (5.6)	793 (33.7)
	40–49	141 (6.0)	24 (1.0)	165 (7.0)
Place of residence	Urban	571 (24.3)	144 (6.1)	716 (30.4)
	Rural	1368 (58.1)	272 (11.6)	1640 (69.6)
Subnational Region	Tigray	216 (9.2)	26 (1.1)	242 (10.3)
	Afar	22 (0.9)	4 (0.2)	26 (1.1)
	Amhara	496 (21.0)	129 (5.5)	625 (26.5)
	Oromia	653 (27.7)	180 (7.6)	832 (35.3)
	Somali	36 (5.5)	4 (1.5)	40 (1.7)
	Benishangul	23 (1.0)	6 (0.2)	28 (1.2)
	SNNPR	401 (17.0)	34 (1.4)	434 (18.4)
	Gambela	9 (0.4)	3 (0.1)	11 (0.5)
	Harari	6 (0.2)	2 (0.1)	7 (0.3)
	Addis Ababa	68 (2.9)	25 (1.0)	93 (3.9)
	Dire Dawa	10 (0.4)	5 (0.2)	15 (0.6)
Cluster altitude in meters	Near sea level	22 (0.9)	5 (0.2)	27 (1.1)
	Low altitude	1083 (46.0)	245 (10.4)	1328 (56.4)
	Moderate altitude	803 (34.1)	155 (6.6)	958 (40.7)
	High altitude	31 (1.3)	12 (0.5)	42 (1.8)
Sex of household head	Male	1679 (71.3)	369 (15.7)	2048 (86.9)
	Female	260 (11.0)	48 (2.0)	307 (13.1)
Age of household head	≤24	109 (4.6)	18 (0.8)	127 (5.4)
	25–39	1115 (47.3)	261 (11.1)	1376 (58.4)
	40–54	534 (22.7)	101 (4.3)	635 (26.9)
	≥55	181 (7.7)	37 (1.6)	218 (9.2)
Marital status	Never in union	10 (0.4)	0 (0.0)	11 (0.5)
	Currently in union/living with a man	1823 (77.4)	394 (16.7)	2216 (94.1)
	Formerly in union/living with a man	106 (4.5)	22 (1.0)	129 (5.5)
Religion	Orthodox	874 (37.1)	182 (7.7)	1056 (44.8)
	Muslim	563 (23.9)	137 (5.8)	700 (29.7)
	Protestant	478 (20.3)	94 (4.0)	571 (24.3)
	Others^a^	24 (1.0)	4 (0.2)	28 (1.2)
Educational level	No education	847 (36.0)	139 (5.9)	987 (41.9)
	Primary	769 (32.7)	188 (8.0)	957 (40.6)
	Secondary	240 (10.2)	48 (0.2)	288 (12.2)
	More than secondary	82 (3.5)	41 (1.8)	123 (5.2)
Literacy	Able to read a whole sentence	1096 (46.5)	213 (9.0)	1309 (55.6)
	Able to read parts of a sentence	257 (10.9)	52 (2.2)	309 (13.1)
	Cannot read at all	569 (24.1)	145 (6.2)	714 (30.3)
	Not assessed	16 (0.7)	7 (0.3)	23 (1.0)
Wealth index	Poorest	276 (11.7)	48 (2.0)	324 (13.7)
	Poorer	383 (16.3)	63 (2.7)	446 (18.9)
	Middle	377 (16.0)	109 (4.6)	486 (20.6)
	Richer	396 (16.8)	59 (2.5)	455 (19.3)
	Richest	506 (21.5)	138 (5.9)	644 (27.4)
Size of family	≤5	1072 (45.5)	262 (11.1)	1333 (56.6)
	>5	868 (36.8)	155 (6.6)	1022 (43.4)

Abbreviation: SNNPR, South Nations and Nationality Peoples Region.

^a^ Others include Catholic, traditional, and other religious denominations.

### 4.2 Obstetrics and reproductive characteristics

About half (50.9%) of women gave childbirth before 18 years old. Over two‐thirds (69.7%) of the women were primiparous in the last 3 years before the survey, respectively. The majority of the respondents (95.6%) had at least three under‐5 children. Similarly, the majority (85.0%) and 1425 (60.5%) of women had at most five living children and were ever born with five or fewer children, respectively. Out of 1782 women who reported preceding birth intervals, more than half (55.2%) of women had at least 37 months (or 3 years) intervals between the most recent births. The birth order number was less or equal to three for 1425 (60.5%) women. The majority (74.2%) of the women reported that they had at least one ANC visit during their most recent pregnancy and more than half (43.3%) had four or more ANC visits. Concerning the place of delivery, nearly half (47.5%) of the most recent childbirths were at home (Table 2).

**TABLE 2:** Maternal reproductive characteristics of the study population, Ethiopia, 2019 (*n* = 2356).

Variables	Description	Adherence to iron tablet intake (weighted)		
		No, *n* (%)	Yes, *n* (%)	Total, *n* (%)
Age of respondent at first birth	<18	978 (41.5)	221 (9.4)	1199 (50.9)
	18–24	809 (34.4)	155 (6.6)	964 (41.0)
	≥25	152 (6.4)	41 (1.7)	193 (8.1)
Total children ever born	≤5	1136 (48.2)	289 (12.3)	1425 (60.5)
	>5	804 (34.1)	127 (5.4)	931 (39.5)
Births in the last 3 years	Nulliparous (0)	475 (20.2)	93 (3.9)	567 (24.1)
	Primiparous (1)	1348 (57.2)	295 (12.5)	1643 (69.7)
	Multiparous (2–3)	117 (5.0)	29 (1.2)	146 (6.2)
Number of ≤5 years children in the household	0	62 (2.6)	14 (0.6)	76 (3.2)
	1 to 3	1859 (78.9)	393 (16.7)	2252 (95.6)
	4–6	19 (0.8)	9 (0.4)	28 (1.2)
Number of living children	≤5	1630 (69.2)	371 (15.8)	2001 (85.0)
	>5	309 (13.1)	45 (1.9)	354 (15.0)
Preceding birth interval [1782]	≤12 months	31 (1.7)	9 (0.5)	39 (2.2)
	13–24 months	229 (12.9)	60 (3.3)	289 (16.2)
	25–36 months	400 (22.4)	69 (3.9)	469 (26.3)
	≥37 months	830 (46.6)	154 (8.6)	984 (55.2)
Birth order number	≤3	1136 (48.3)	289 (12.3)	1425 (60.6)
	4–6	558 (23.7)	98 (4.2)	656 (27.9)
	≥7	246 (10.4)	29 (1.2)	275 (11.6)
Antenatal care checkup during most recent pregnancy	No	108 (4.6)	6 (0.3)	115 (4.9)
	Yes	1831 (77.7)	410 (17.4)	2241 (95.1)
Frequency of antenatal care visits (1939)	No visit	100 (4.2)	6 (0.3)	106 (4.5)
	1–3	764 (32.5)	128 (5.4)	893 (37.9)
	≥4	1075 (45.6)	282 (12.0)	1357 (57.6)
Timing of first antenatal visits [2249]	First trimester	1143 (50.8)	320 (14.2)	1463 (65.0)
	Second trimester	628 (27.9)	88 (3.9)	715 (31.8)
	Third Trimester	69 (3.1)	2 (0.1)	71 (3.2)
Past place of delivery	Homes	665 (28.3)	107 (4.5)	772 (32.8)
	Health facilities	1274 (54.1)	310 (13.1)	1584 (67.2)

Women who had five or fewer live children and who had received antenatal care (ANC) visits during their most recent pregnancy adhered to iron intake in proportions of 371 (15.8%) and 410 (17.4%), respectively. Similarly, 221 (9.4%), 295 (12.5%), and 289 (12.3%) women whose age at first birth is less than 18, primiparous women, and whose birth order number was three or less have adhered to iron intake. Additionally, 282 (12.0%) and 320 (14.2%) women who had at least four ANC visits during their first trimester adhered to iron intake, respectively (Table 2).

### 4.3 The proportion of adherence to iron supplement intake during pregnancy

**FIGURE 1:**
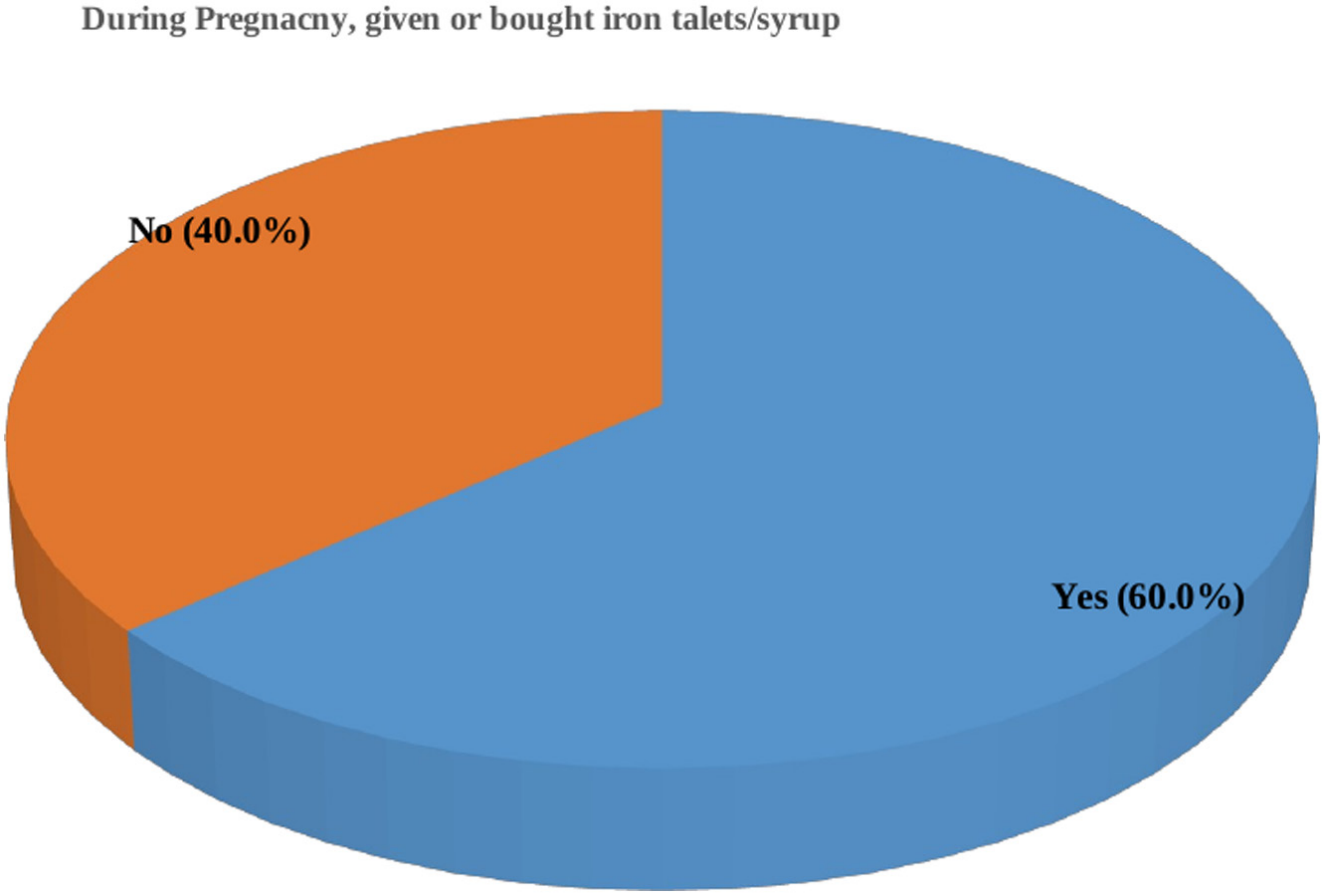
The proportion of pregnant women who took iron supplements in Ethiopia, 2019 (*n* = 3927).

**FIGURE 2:**
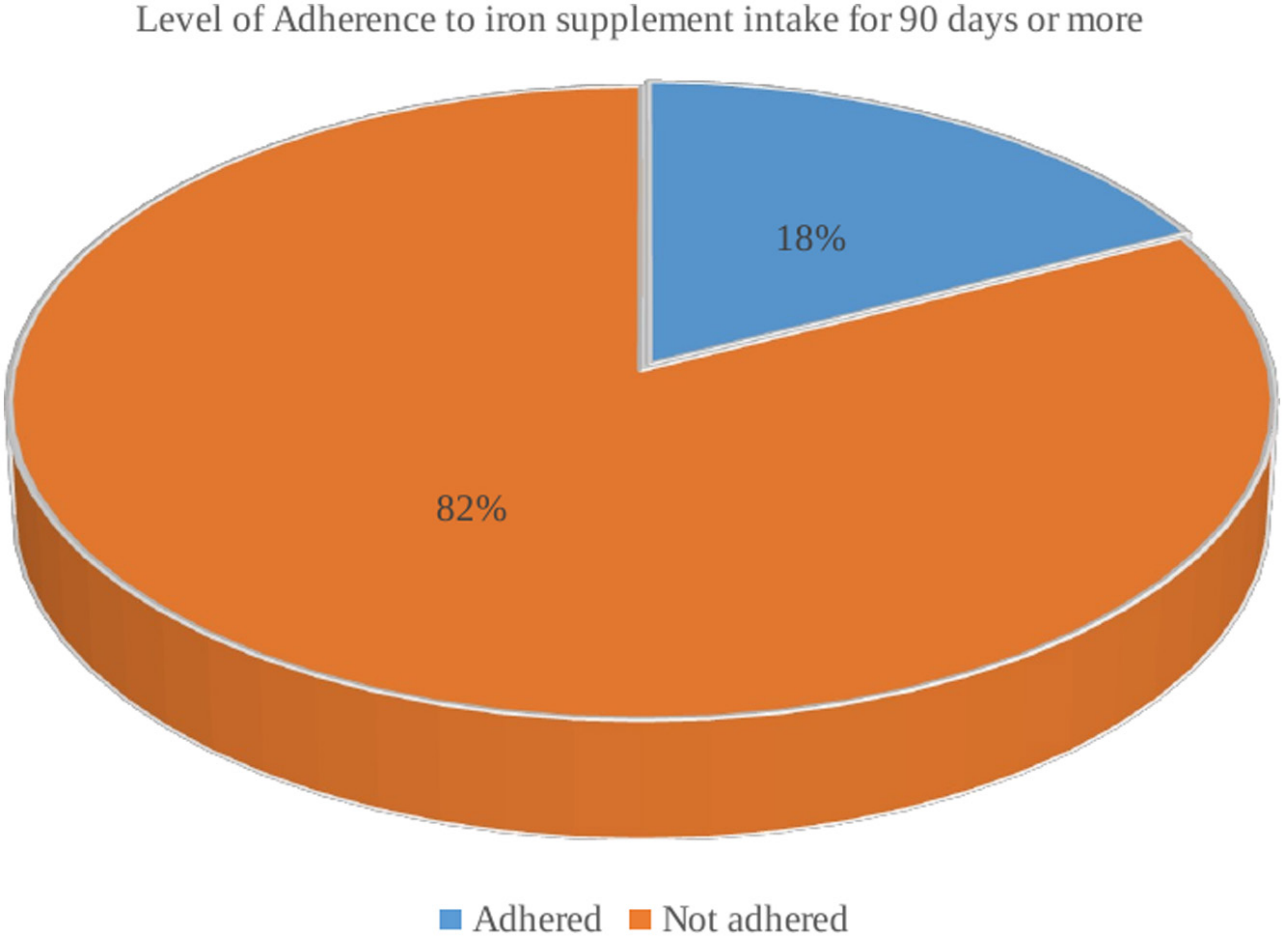
The proportion of pregnant women who adhered to iron supplement intake in Ethiopia, 2019 (*n* = 2356).

In general, 2356 (60.0%) of the women in this study took iron supplements during their most recent pregnancy (Figure 1). However, only 417 (17.7%; 95% CI: 0.162–0.193) of the women who received iron supplements continued to take them for at least 90 days (Figure 2). Only a negligible number, 14 (0.6%), of women had taken the iron supplements for 60–89 days, whereas more than three‐fourths (79.2%) had only used them for 60 days or fewer. In addition, 60 (2.5%) of the pregnant women said they had not taken iron or were unsure of whether they had taken it.

### 4.4 Factors associated with adherence to iron intake

Subnational region of respondents, highest educational level, literacy, the timing of the first ANC booking, and place of the most recent delivery (childbirth) were all linked to adherence to the use of iron supplements in multivariable logistic regression. While literacy and the timing of the first ANC booking were negatively correlated with the outcome variable, the respondent's subnational region, highest educational level, and last place of delivery were positively associated.

The odds of adherence to iron supplement intake were nearly three times higher among those women living in Amhara [AOR = 2.71; 95% CI: 1.69, 4.34] and Oromia regions [AOR = 2.78; 95% CI: 1.75, 4.41] as compared to women from Tigray region. Similarly, the odds of adherence to iron supplement intake were nearly two times higher among those women living in Addis Ababa city administrations [AOR = 2.37; 95% CI: 1.34, 4.53] and more than three times higher among women living in Dire Dawa city administrations [AOR = 3.44; 95% CI: 1.02, 11.65] as compared to women from Tigray region (Table 3).

**TABLE 3:** Bivariate and multivariable analysis of adherence to iron tablets/syrup intake during pregnancy among women who gave birth in the last 5 years in Ethiopia, 2019 (*n* = 2356).

Variables	Description	COR (95% CI)	AOR (95% CI)
Place of residence	Urban	1	1
	Rural	0.79 (0.63, 0.99)*	0.92 (0.63, 1.33)
Subnational region	Tigray	1	1
	Afar	1.69 (0.56, 5.10)	1.96 (0.62, 6.23)
	Amhara	2.19 (1.36, 3.45)***	2.71 (1.69, 4.34)***
	Oromia	2.32 (1.49, 3.60)***	2.78 (1.75, 4.41)***
	Somali	0.92 (0.30, 2.80)	0.56 (0.13, 2.43)
	Benishangul	2.09 (0.76, 5.71)	1.86 (0.66, 5.23)
	SNNPR	0.70 (0.41, 1.22)	0.87 (0.49, 1.53)
	Gambela	2.89 (0.71, 11.52)	1.38 (0.26, 7.31)
	Harari	2.86 (0.52, 15.75)	2.77 (0.47, 16.33)
	Addis Ababa	3.03 (1.63, 6.61)***	2.37 (1.34, 4.53)***
	Dire Dawa	3.85 (1.19, 12.48)*	3.44 (1.02, 11.65)*
Highest educational level	No education	1	1
	Primary	1.48 (1.17, 1.89)***	1.54 (1.09, 2.20)**
	Education	1.21 (0.85, 1.73)***	1.48 (0.87, 2.23)
	More than secondary	3.06 (2.02, 4.63)	3.49 (1.91, 6.36)***
Literacy	Cannot read at all	1	1
	Able to read a whole sentence	1.32 (1.04, 1.67)*	0.59 (0.40, 0.87)**
	Able to read parts of a sentence	1.04 (0.74, 1.44)	0.66 (0.44, 0.99)*
	Not assessed	2.06 (0.82, 5.15)	3.44 (0.88, 13.36)
Wealth index	Poorest	1	1
	Poorer	0.95 (0.63, 1.43)	0.83 (0.54, 1.28)
	Middle	1.68 (1.15, 2.44)**	1.36 (0.91, 2.06)
	Richer	0.89 (0.58, 1.31)	0.63 (0.40, 1.00)
	Richest	1.59 (1.12, 2.28)**	0.88 (0.52, 1.48)
Family size	≤5	1	1
	>5	0.73 (0.59, 0.91)**	1.05 (0.77, 1.42)
Number of living children	≤5	1	1
	>5	0.65 (0.46, 0.90)**	0.99 (0.65, 1.52)
Total children ever born	≤5		
	>5	0.62 (0.50, 0.78)***	0.75 (0.53, 1.07)
Birth order	≤3	1	1
	4 to 6	0.69 (0.54, 0.89)**	
	≥7	0.47 (0.31, 0.70)***	
ANC checkup	No	1	1
	Yes	3.73 (1.68, 8.30)***	
Number of ANV visits	No visit	1	1
	1–3 visits	2.58 (1.14, 5.83)*	
	≥4 visits	4.02 (1.80, 9.00)***	1.25 (0.97, 1.61)
Timing of first ANC visits	First trimester	1	1
	Second trimester	0.50 (0.39, 1.69)***	0.63 (0.48, 0.84)***
	Third Trimester	0.11 (0.03, 0.43)**	0.12 (0.03, 0.50)**
Place of delivery	Homes	1	1
	Health facilities	1.52 (1.19, 1.92)***	1.37 (1.04, 1.81)*
*Goodness‐of‐fit test*			
*Hosmer and Lemeshow test*		*p‐value = .4039*	
*Likelihood Ratio Test*		*125.93*	

Abbreviations: ANC, antenatal care; AOR, Adjusted odds ratio; CI, confidence interval; COR, crude odds ratio; SNNPR, South Nations and Nationality Peoples Region.

*Significant *p*‐value <.05, **significant *p*‐value <.01; ***Significant *p*‐value <.001.

Likewise, the odds of adherence to iron were about two times higher among those women with primary education [AOR = 1.54; 95% CI: 1.09, 2.20] and more than three times higher among women with more than secondary education [AOR = 3.49; 95% CI: 1.91, 6.36] as compared to women with no education. Women who were able to read the whole sentence were 41% [AOR = 0.59; 95% CI: 0.40, 0.87] and those who were able to read parts of a sentence were 34% [AOR = 0.66; 95% CI: 0.44, 0.99] less likely to adhere to iron supplement intake as compared to women who cannot read at all (Table 3).

Moreover, women who booked their first ANC during the second [AOR = 0.63; 95% CI: 0.48, 0.84] and third trimesters [AOR = 0.12; 95% CI: 0.03, 0.50] were 39% and 88% less likely to adhere to iron supplements for 90 days or more as compared to women who booked their first ANC during the first trimester. Furthermore, the odds of adherence to iron supplement intake were 1.37 times higher among women who had delivered at any health facility [AOR = 1.37; 95% CI: 1.04, 1.81] than those who gave childbirth at home (Table 3).

## 5 DISCUSSION

Based on the 2019 EMDHS data, this analysis calculated the percentage of adherence to iron supplement intake during pregnancy and identified factors associated with it in Ethiopia. Our findings showed that the majority of respondents (60%) took iron supplements during their most recent pregnancy, but only 17.7% (95% CI: 0.162–0.193) of pregnant women maintained a 90‐day or longer iron supplement regimen. This finding was consistent with a previous study in Ethiopia (Haile et al., 2017). However, adherence to iron supplement intake in this study is lower compared with the findings of other similar studies (no nationwide study) elsewhere in Ethiopia (Boti et al., 2018; Getachew et al., 2018; Tamirat et al., 2022; Taye et al., 2015), and findings of the study in Germany (Demuth et al., 2018), Khartoum Sudan (Abdullahi et al., 2014), Malawi (Titilayo et al., 2016), North‐Western Tanzania (Lyoba et al., 2020), and North West province of South Africa (Mbhenyane & Cherane, 2017). This finding is also lower than the systematic review and meta‐analysis conducted in Ethiopia, in which the overall pooled prevalence of adherence to iron and folic acid supplementation among pregnant women in Ethiopia was 41.38% (Sendeku et al., 2020).

The inconsistency could be related to geographical and socio‐economical differences. The differences may be due to most of the previous studies conducted in Ethiopia were relatively using a small sample of pregnant women, while EMDHS was conducted on a nationally representative sample. There are also variations in the time of the study and study setting. The high prevalence of iron deficiency anemia and the low adherence to iron supplementation recommendations contribute substantially to the high morbidity and mortality levels among women of reproductive age in Ethiopia. As a result, further interventions are needed to increase adherence to iron/folic acid supplement intake, which is low in progress, considering the global strategy to reduce anemia by 2025.

While respondents' subnational region, highest educational level, and last place of delivery were positively associated with the outcome variable, literacy and timing of first ANC booking were negatively associated with the outcome variable.

In this study, women living in some subnational regions (Amhara and Oromia regions, and Addis Ababa and Dire Dawa cities administrations) had higher odds of adhering to iron intake than women from the Tigray region. This may be attributed to sociodemographic and agroecological variations. Women from Addis Ababa and Dire Dawa cities may have more information, be more educated, have more exposure to media, and have more access to healthcare services than those from the Tigray region. However, the discrepancies between the Tigray region and Amhara and Oromia regions have to be investigated although there are variations among the study populations in terms of living standards, nutritional information, and access to health care services among these regions.

This study revealed that women's educational status was positively associated with adherence to iron intake. That is the proportion of women taking iron for 90 days or more increases with increasing education. The odds of adherence to iron intake were higher among those women with primary education and more than secondary education as compared to women with no education. Similarly, this result is in line with the findings of other studies; showing that the educational status of secondary school and above was significantly associated with good adherence to IFA supplementation as revealed by previously published literature from Ethiopia and Malawi (Agegnehu et al., 2019; Boti et al., 2018; Gebreamlak et al., 2017; Tarekegn et al., 2019; Tegodan et al., 2021; Titilayo et al., 2016). The finding is supported by other studies done in the Mecha district, Northwest Ethiopia, Indonesia, and West Iran (Rialine & Dibley, 2015; Sendeku et al., 2020; Taye et al., 2015) (Zhou et al., 2017).

Educated women are likely to have better knowledge and access to information through reading and understanding about iron deficiency anemia and therapy, the benefits of supplements, and pregnancy in general. Third, it might be associated with the notion that education is more likely to enhance female awareness of micronutrient deficiency and ways to overcome these deficiencies. Secondly, it might be associated with the fact that educated women have a greater ability to stick to health care inputs such as IFA, which offer better care for both the infant and the mother. The results reinforce the need for improvements in the education of women of reproductive age in Ethiopia, a few of the women had secondary (12.2%) and more than secondary (5.2%) education and 41.9% had no education.

Women who were able to read the whole sentence were 41% and those who were able to read parts of a sentence were 34% less likely to adhere to iron supplements for 90 days or more as compared to women who cannot read at all. This may be because those women who cannot read might not read the side effects of iron and be exposed to different views concerning iron consumption, which can inhibit them to consume it. There is no previous study that showed adherence to iron was negatively associated with literacy.

Moreover, this study predicts the timing of first ANC visits correlated negatively with adherence. Women who booked their first ANC during the second and third trimesters were 39% and 88% less likely to adhere to iron supplements for 90 days or more as compared to women who booked their first ANC during the first trimester. Similar findings were reported by studies conducted elsewhere in Ethiopia and other countries (Agegnehu et al., 2019; Boti et al., 2018; Digssie Gebremariam et al., 2019; Gebre et al., 2015; Lyoba et al., 2020; Sendeku et al., 2020; Titilayo et al., 2016). The possible justification might be pregnant women who registered early for ANC service could acquire a better knowledge of the perceived risk and benefits of IFAS to prevent anemia during pregnancy. This might affect directly the duration and number of tablets consumed to adhere to the IFAS program. Furthermore, the odds of adherence to iron supplements for 90 days or more were 1.37 times higher among women who had delivered at any health facility than those who gave childbirth at home. This might be due to health care providers in charge of ANC and delivery services may counsel women on the benefit of taking the supplement and nutrition at the right time and dose by discussing adherence benefits and consequences of nonadherence for the mother and the fetus, which ultimately improves the adherence status of pregnant women to the supplement. Moreover, at governmental health institutions, iron is freely supplemented. Thus, depending on their health status, they could be informed about the prevention of anemia through iron supplementation.

In addition, other studies in Ethiopia and other countries indicated two or more (four or more in some studies) ANC visits and numbers of ANC visits were positively associated with IFA adherence (Ba et al., 2019; Demis et al., 2019; Gebre et al., 2015; Kiwanuka et al., 2017; Sendeku et al., 2020; Solomon et al., 2021; Tarekegn et al., 2019) though it did not associate with the outcome variable in this study. This is the fact that ANC is the vital route for the delivery of iron supplementation and reinforcement of adherence. This suggests that increased ANC visits are a good opportunity to increase contact between pregnant women and health professionals. Thus, health professionals can disseminate key information/messages, especially the benefits of IFA supplementation.

Nonetheless, women's age, age of the HH head, sex of the HH head, place of residence, cluster altitude, religion, ethnicity, wealth index, family size, age of respondent at first birth, births in the last 3 years, number of totals and living children, birth order, number of under‐five children, ANC check‐ups, frequency of ANC visits and birth interval did not associate with adherence of iron supplement intake in this study.

The study used more recent, large, and nationally representative data which also take into account all regions of Ethiopia. In addition, this study was conducted in the community and followed all DHS data rules, including weighting. These enhance the generalizability of the findings. However, this study is not without limitations. First, this analysis has relied solely on secondary data. Similarly, some variables (hemoglobin and level of anemia) were excluded from the analysis because of missing values. Some pregnancy and health‐related factors, supplement‐related factors, and knowledge of anemia were not included in the study. Further, the data, self‐reported by the study participants, was susceptible to recall bias. The estimation of adherence to iron supplement intake based on the self‐report method may underestimate the prevalence of nonadherence when compared with objective measures like pill counts or biological assays and medication adherence measures.

## 6 CONCLUSIONS

The results indicate very low adherence to iron in Ethiopia compared to the World Health Organization's iron supplementation recommendations among pregnant women. While respondents' subnational region, highest educational level, and last place of delivery were positively associated with the outcome variable, literacy and timing of first ANC booking were negatively associated with the outcome variable. These results underscore the need for increased efforts to improve the uptake of iron supplementation for pregnant women in Ethiopia. Focusing on interventions that target populations with low rates of iron supplement intake, including early initiation and frequent antenatal care visits, institutional delivery, and raising community awareness through educational programs for pregnant women are recommended to improve adherence to iron intake. Therefore, health professionals and responsible bodies should give attention to advising and counseling a pregnant woman on the benefits and starting time of iron folate supplementation.

## AUTHOR CONTRIBUTIONS

**Takele Gezahegn Demie:** Conceptualization (lead); data curation (lead); formal analysis (lead); methodology (equal); resources (lead); software (lead); validation (equal); writing – original draft (lead); writing – review and editing (lead). **Simegnew Handebo:** Conceptualization (supporting); data curation (supporting); formal analysis (supporting); methodology (equal); resources (supporting); software (supporting); validation (supporting); writing – review and editing (equal). **Getachew Tilahun Gessese:** Conceptualization (supporting); data curation (supporting); formal analysis (supporting); methodology (equal); resources (supporting); software (supporting); validation (supporting); writing – review and editing (supporting). **Berhanu Teshome Woldeamanuel:** Conceptualization (supporting); data curation (supporting); formal analysis (supporting); methodology (supporting); resources (supporting); software (supporting); validation (supporting); writing – review and editing (supporting). **Tolesa Diriba Biratu:** Conceptualization (supporting); data curation (supporting); formal analysis (supporting); methodology (lead); resources (supporting); software (supporting); validation (lead); writing – review and editing (lead).

## FUNDING INFORMATION

The authors received no specific funding for this work.

## CONFLICT OF INTEREST STATEMENT

The authors declare that they have no conflict of interest.

## ETHICS STATEMENT

The study used data from the 2019 EMDHS. The Federal Democratic Republic of Ethiopia's Ministry of Science and Technology and the Institutional Review Board of ICF International reviewed and approved the survey protocol. It was conducted according to the Declaration of Helsinki and all respondents gave their informed consent prior to their inclusion in the study. As a result, specific ethical approval and consent to participate in the study are not applicable.

## PATIENT CONSENT STATEMENT

NA.

## Data Availability

The data that support the findings of this study are available on request or upon registration from the DHS website: http://www.dhsmeasures.
